# Peptides Labeled with Pyridinium Salts for Sensitive Detection and Sequencing by Electrospray Tandem Mass Spectrometry

**DOI:** 10.1038/srep37720

**Published:** 2016-11-28

**Authors:** Mateusz Waliczek, Monika Kijewska, Magdalena Rudowska, Bartosz Setner, Piotr Stefanowicz, Zbigniew Szewczuk

**Affiliations:** 1 Faculty of Chemistry, University of Wrocław, F. Joliot-Curie 14, 50-383 Wrocław, Poland

## Abstract

Mass spectrometric analysis of trace amounts of peptides may be problematic due to the insufficient ionization efficiency resulting in limited sensitivity. One of the possible ways to overcome this problem is the application of ionization enhancers. Herein we developed new ionization markers based on 2,4,6-triphenylpyridinium and 2,4,6-trimethylpyridinium salts. Using of inexpensive and commercially available pyrylium salt allows selective derivatization of primary amino groups, especially those sterically unhindered, such as ε-amino group of lysine. The 2,4,6-triphenylpyridinium modified peptides generate in MS/MS experiments an abundant protonated 2,4,6-triphenylpyridinium ion. This fragment is a promising reporter ion for the multiple reactions monitoring (MRM) analysis. In addition, the fixed positive charge of the pyridinium group enhances the ionization efficiency. Other advantages of the proposed ionization enhancers are the simplicity of derivatization of peptides and the possibility of convenient incorporation of isotopic labels into derivatized peptides.

Elucidation of processes taking place in living cells, especially interactions of diverse range of biomolecules, is an object of widespread interest^1^,^2^. In this diversity of biomolecules particularly important is an analysis of protein whose dysfunction leads to many of diseases^3^,^4^,^5^,^6^. Identification of proteins and the insight into their interactions as well as determination of post-translational modifications contributes to understanding of their biological function or disease state^7^. Quantitative proteomic analysis has been rapidly growing field that uses mass spectrometry as a leading analytical tool allowing for example to monitor of protein or peptides concentrations^8^,^9^. Despite its great potential documented in literature, numerous applications as well as increasingly sophisticated technology of mass spectrometers, there are limitations in analysis of trace amounts of peptides resulting from insufficient ionization efficiency which limits the sensitivity during analysis by mass spectrometry.

ESI-MS is one of the mild ionization techniques that allow to observe multiply charged compounds, however to enable the detection a necessity of in-solution ionization is associated with this technique. The proton affinity is an important parameter influencing the intensity, which in turn depends not necessarily on the concentration, but on the specific characteristics of a peptide sequence, hence abundant signals of Arg-containing peptides in ESI spectra^10^. Another way to enhance ionization efficiency of peptides is their derivatization which results in a formation of fixed charged peptide derivative and their increased intensity in ESI^11^,^12^. Several derivatization strategies leading to the formation of charge-tagged peptides are reported including quaternary ammonium (QAS)^13^ or phosphonium salt (QPS)^14^.

Recently, we developed a method allowing to obtain peptide conjugates containing *N,N,N*-trialkyl moieties^15^,^16^. Although this method was characterized by improved sensitivity of detection, even at the attomole level^17^, their applicability to peptide sequencing was questionable due to the insufficient stability of linear *N,N,N*-trialkyl QAS, during MS/MS experiment in view of Hofmann elimination^18^. Due of these reasons, QAS-peptide conjugates based on rigid amines such as 1-azabicyclo[2.2.2] octane (ABCO)^19^ and 1,4-diazabicyclo[2.2.2]octane (DABCO)^20^ or 5-azaspiro[4.4]nonane^21^ was developed to eliminate of the quoted problem.

Pyrylium salts have been known since 100 years^22^, but there has been an increased interest in them over forty years ago due to their importance as reactive intermediates for synthesis of heterocyclic compounds. Pyrylium cations are highly reactive toward nucleophiles; therefore they react readily with primary amines to give pyridinium derivatives^23^. Several routes of pyrylium salts synthesis were proposed^24^,^25^. Data presented in literature demonstrated that pyrylium salts may be applied for modification of proteins^26^. The high reactivity of these compounds and their selectivity toward ε-amino groups of lysine makes them interesting reagents for incorporation of fluorophores into biomolecules. However, the utility of pyridinium salts as an ionization enhancer has not been reported yet.

Here, we present a new approach of peptide derivatization based on conversion of ε-amino groups of lysine side chain to trisubstituted pyridinium derivative. This derivatization results in a highly improved sensitivity of peptide detection. In this study we used 2,4,6-triphenylpyrylium and 2,4,6-trimethylpyrylium salts as ionization tag reagents. These compounds are commercially available, inexpensive and can be easily prepared with a good yield by a one-pot cyclization of e.g. two equivalents of benzalacetophenone and one equivalent of benzaldehyde^27^,^28^,^29^. The proposed approach is particularly useful for analysis of tryptic peptides containing C-terminal lysine residue. In contrast to commonly used ammonium or phosphonium salts, which require conjugation with reactive groups like e.g. succinimide esters, pyrylium salts are itself reactive toward primary amino groups which significantly simplify the derivatization^30^.

The presence of a fixed charge at C-terminus causes charge remote fragmentation^31^ upon collisional activation in MS/MS experiment resulting in observation of mainly *y* series (according to nomenclature of Roepstorff and Fohlman^32^, with a high sequence coverage, so that the analysis becomes facilitated and predictable. We investigated mechanism of elimination of the substituted pyridinium cation from labeled peptides and discussed utility of this fragment as a reporter ion in multiple reaction monitoring. We also presented the straightforward and cost-effective synthesis of deuterated isotopologues. This fact, combined with the elimination of the pyridinium reporter ion, enables highly sensitive MRM-based analysis, which is potentially useful in quantitative proteomics.

## Results and Discussion

Mass spectrometry analysis of trace amounts of peptides is problematic due to the insufficient ionization efficiency, which results in limited sensitivity. One of a possible ways to overcome this limitation is application of ionization enhancers. Herein we describe an application of 2,4,6-trisubstituted pyrylium salts as reagents allowing incorporation of pyridinium moiety to amino group of a peptide and studies of the effect of this modification on the ionization efficiency. The model reactions were performed on a solid support by derivatization of N-terminal amino group in peptides. After the synthesis of a peptide on the a solid support according to the Fmoc strategy, the Fmoc group was removed in a presence of piperidine and then a reaction between the peptide and an excess (3 eq.) of 2,4,6-triphenylpyryllium or 2,4,6-trimethylpyrylium salt was performed. The derivatization reaction proceeds in two stages presented in Fig. S3 in Supporting Information. The first step requiring alkaline conditions was conducted in the presence of triethylamine while the second one was carried out in the presence of acetic acid which catalyzes the reaction. Alternatively, the resin-bound peptides, containing MTT (4-methyltrityl group) protected Lys residue were deprotected using 1% TFA /DCM and then modified using the pyrylium salts. The excess of derivatizing agent was removed by washing the resin 5 times with DMF and then 3 times with dichloromethane and methanol.

The examples of chromatograms and MS spectra of selected pyrylium salts synthesized on solid support are presented on Fig. S4. The spectrum is dominated by the signal at *m/z* 309.124 corresponding to the desired product. The purity of this compound was confirmed by HPLC. The synthetic 2,4,6-trimethylpyrylium salt (TPP) was used for derivatization of model peptides on a solid support. The representative spectrum of the result of this synthesis is presented on Fig. S5. Only one signal at m/z 513.2 corresponding to desired product is observed on the spectrum. Our investigation has demonstrated that this reaction allows the convenient derivatization of primary peptide amino groups, especially these sterically unhindered, such as glycine and alanine or ɛ-amine group of lysine. The other amino acids residues did not give the desired product after derivatization. Obtained model compounds were used in further investigations of peptides modified with pyrylium salts including CID fragmentation and chromatographic properties.

### Collision induced dissociation of peptides modified with pyrylium salts

**Figure 1:**
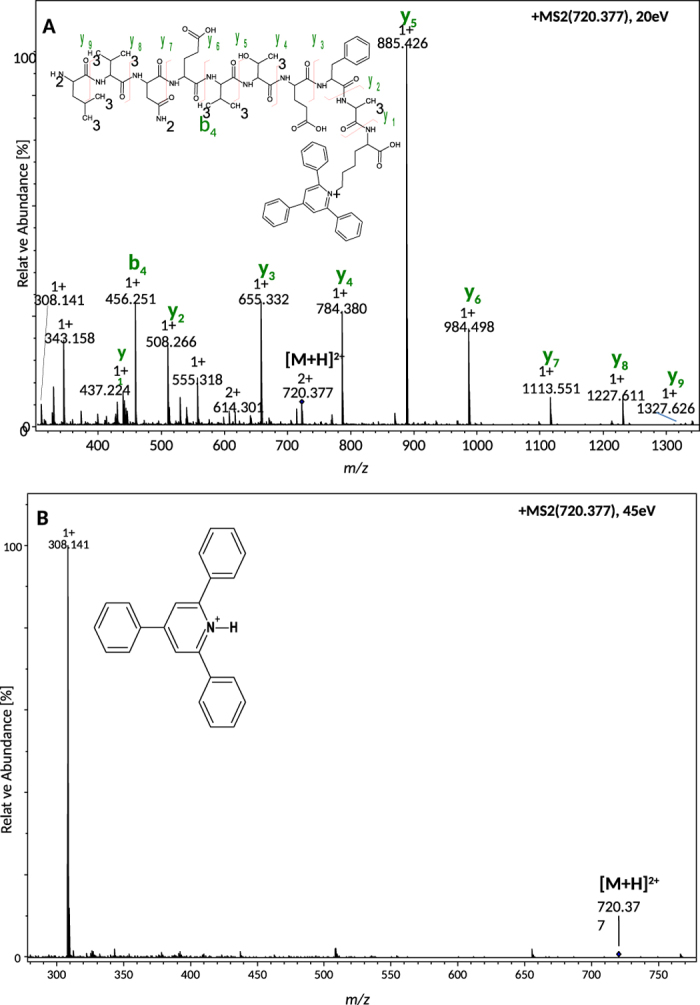
ESI-MS/MS spectrum for derivatized tryptic peptide LVEQVEFAK^#^(TPP^+^) (**A**) CID 20 eV, (**B**) 45 eV (K^#^ - lysine residue without the amino group at ɛ-amino group).

Selected modified peptides were subjected to CID MS/MS experiments at various collision energies. The representative spectra for this experiment are presented in Fig. S6 and Fig. 1. The fragmentation spectrum of TPP^+^-Gly^#^-Phe-OH (where # denotes deamino) at collision potential of 20 V (Fig. S6) is dominated by fragment peak at *m/z* 308.139 corresponding to protonated 2,4,6-triphenylpyridinim ion.

The CID experiment was performed on 2+ ion of tryptic (M+2 H)^2+^ peptide LVEQVEFAK^#^(TPP^+^) in which the ɛ-amino group of C-terminal lysine residue was substituted by 2,4,6–triphenylpyridynium group. A moderate collision potential of 20 V was applied. The fragmentation gave rise to the abundant series of y* type of ions only (denotes y-H), which facilitates the sequencing of peptide (Fig. 1A). Contrary to lower collision energy, application of higher collision energy (45 eV) during the experiment revealed the intensive signal of a diagnostic ion at *m/z* 308.141 (Fig. 1B). This strong diagnostic fragment ion may be useful in analysis of tryptic digests containing lysine residue on C-terminus in analysis of trace amounts of proteins via LC-MS/MS in multiple reaction monitoring analysis (MRM) mode.

The fragmentation analysis of peptides derivatized with 2,4,6-trimethyl pyrylium ions revealed formation of analogous, protonated 2,4,6-trimethylpyridinium ion at *m/z* 122.096 (Fig. S7). High intensity of these ions, especially at high collision energy, makes them promising reporter ions for multiple reactions monitoring analysis (MRM). In the spectra of N-terminally modified peptides the peak corresponding to protonated 2,4,6–triphenyl- or 2,4,6-trimethyl–pyridinium ion is accompanied by ions at *m/z* 320.130 and 137.116, respectively. Abundancies of these ions increases with collision potential in CID experiment. The peaks at *m/z* 320.130 and 137.116 were not observed in MS/MS spectra of peptides modified on lysine moiety.

In order to elucidate the structures of these ions three different isotopically labeled analogues of precursor peptides were prepared using two distinct protocols. The first one concerned the solution phase synthesis of isotopically labeled 2,4,6-triphenylpyrylium salt starting from deuterated benzene as a substrate while the next two was based on H/D exchange in the derivatized peptide. In the first procedure we synthesized 2,4,6-triphenylpyrylium salt deuterated in two phenyl rings (D_10_). The reaction was carried out according the protocol described by Dimroth^29^ (see Experimental Section). Briefly: After the Friedl-Crafts acylation of benzene-d_6_ with acetic anhydride the aldol condensation of formed acetophenone-d_5_ and benzaldehyde was performed yielding the benzalacetophenone-d_5_. The further condensation of benzalacetophenone-d_5_ and acetophenone-d_5_ in a presence of trifluoromethansulphonic acid results in formation of 2,4,6-triphenylpyryllium-d_10_ salt with the trifluoromethansulphonate as a counterion. The product was synthesized with high yield. The identity and purity of this compound was confirmed by ESI-MS and HPLC, respectively (Fig. S8). In Fig. S9 ESI-MS spectra of TPP^+^-Gly^#^-Ala-Phe-Gly-NH_2_ and its isotopologue (TPP^+^-Gly^#^-Ala-Phe-Gly-NH_2_-d_10_) are presented. As it was expected, the molecular mass of the labeled compound is shifted by 10 mass units, as compared to its unlabeled counterpart.

**Figure 2:**
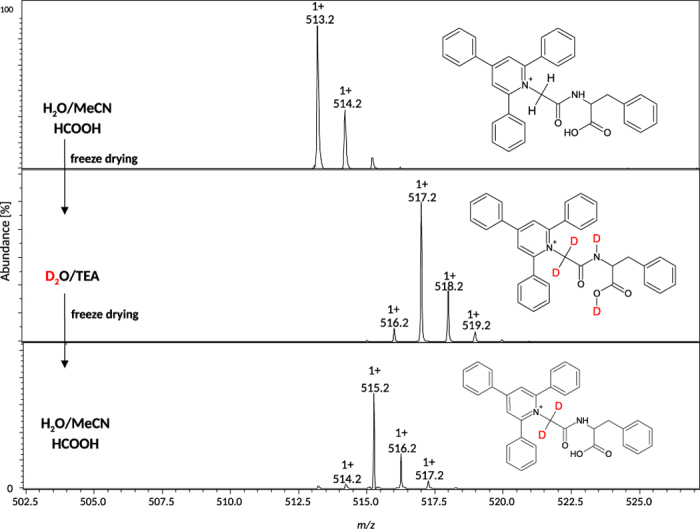
ESI-MS spectra of TPP^+^-Gly^#^-Phe-OH before and after incubation in D_2_O/TEA.

The second procedure was based on the H/D exchange of α-C hydrogens described previously for non-aromatic betaine derivatives (*N,N,N*,-trialkyloglycine)^33^. This method is limited to peptides containing the quaternary salt at the N-terminal α-amino acids, but cannot occur on the lysine side chain. The deuterated isotopologues of peptides containing TPP^+^-Gly^#^ residue were obtained through incubation of peptides in heavy water containing 1% of trimethylamine (TEA), over 10 min at room temperature. Under these conditions, the peptide exchanges 6 protons, but after lyophilization followed by the back exchange in water at pH = 4, only two deuterium atoms at alpha carbon of glycine remain. The corresponding mass spectra presented in Fig. 2 show that the peptides containing TPP^+^-Gly^#^- residue may undergo the H/D exchange of α-C hydrogens in a presence of 1% TEA, similarly to non-aromatic betaine derivatives.

Another experiment was performed for peptides containing trimethylpyridinium salt. To obtain the deuterated isotopologue the modified peptide was incubated for 24 h in heavy water containing 1% of TEA at 50 °C. Then, the sample was lyophilized followed by dissolving in water with addition of acetonitrile for complete dissolving. Eleven of the hydrogen atoms were exchanged for deuterium: 9 from the three methyl groups and 2 from α carbon. The obtained MS spectrum and structures of deuterated compounds is presented in Fig. S10.

**Figure 3:**
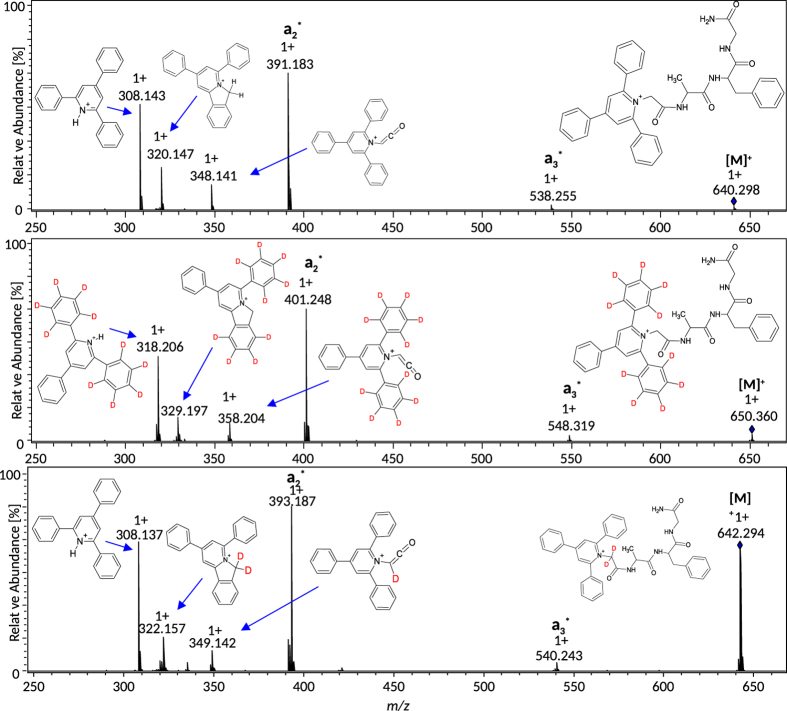
CID fragmentation of TPP^+^-Gly^#^-Ala-Phe-Gly-NH_2_ and its deuterated analogues.

The signals corresponding to isotopologues of 2,4,6-trisubstituted pyrylium salt (650.363; 640.303; 642.322 *m/z*) were subjected to CID fragmentation (Fig. 3). The analysis of ESI-MS/MS spectra of a peptide containing deuterated (D_2_ and D_10_) triphenylpyridinium moiety allow to propose the structures for the two most important diagnostic ions formed during these experiments (for non-labeled compound: 308.14 and 320.15 *m/z*). The analysis of fragmentation of D_2_ isotopologue reveals formation of fragments at *m/z* 308.14 (0 D) and 322.14 (2 D) while fragmentation of D_10_ isotopologue gives a rise to fragments at *m/z* 318.20 (10 D) and 329.20 (9 D) respectively. The number of deuterium atoms in product ions suggests that fragment at m/z 308.14 is a protonated pyridinium ion while ion at m/z 320.15 contains a five-membered ring formed by bridging a nitrogen atom with C2 carbon in a phenyl ring. Other signals in spectra presented below were identified as a typical fragments appearing from the cleavage of peptide bonds. In these cases the series of a ions are observed.

Similar fragmentation analysis was conducted for labeled and non-labeled trimethylpyridinium salt. The undeuterated isotopologue form the product ions at m*/z* 122.09 corresponding to protonated pyridinium ion which is analogous to the one at *m/z* 308.14 formed from TPP derivatives and fragment at m/z 137.12. Interestingly, according to nitrogen rule, the last ion has odd number of electrons. The number of deuterium atoms in product ions obtained by fragmentation of D_11_ isotopologue of 2,4,6-trimethylpyridinium derivative of Gly-Phe peptide (9 and 11 deuterium atoms for signals at m/z 131.15 and 146.19, respectively) allowed proposing for the fragment ion at m/z 137.12 the cation-radical type structure presented in Fig. S11 however other isomers cannot be excluded.

### Influence of isotopic substitution on chromatographic properties of peptides derivatized with pyrylium salts

**Figure 4:**
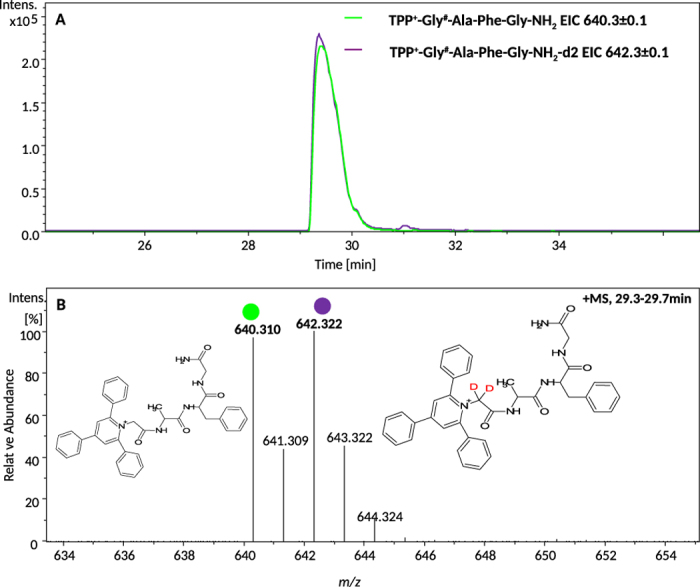
LC-MS analysis for the compounds TPP^+^-Gly^#^-Ala-Phe-Gly-NH_2_ and TPP^+^-Gly^#^-Ala-Phe-Gly-NH_2_-d_2_. (**A**) Extracted ion chromatograms for the ions at 640.3 *m/z* and 642.3 *m/z* corresponding to TPP^+^-Gly^#^-Ala-Phe-Gly-NH_2_ and TPP^+^-Gly^#^-Ala-Phe-Gly-NH_2_-d_2_ respectively; (**B**) ESI-MS spectrum for compounds eluted at retention time 29.3–29.7.

We investigated also the impact of isotope substitution on the retention time of the peptides derivatized with pyrylium salts. Isotopically labeled compounds are routinely used for quantitative analysis of complex mixtures. According to literature, deuterated compounds are in many cases subject to chromatographic shifts in respect to the unlabeled compound, which may cause significant quantification errors. The LC-MS analysis of equimolar mixture of a non-labeled peptide and its isotopologue containing 2 deuterium atoms at α carbon (TPP^+^-Gly^#^-Ala-Phe-Gly-NH_2_-d_2_) indicated that there is no significant difference in their retention times. (Fig. 4). Additionally the analysis of peak purity presented in Fig. S12 confirmed the co-elution of compounds.

On the other hand, the LC-MS analysis of isotopically labeled (TPP^+^-Gly^#^-Ala-Phe-Gly-NH_2_-d_10_) and non-labeled peptides showed significant shift of the chromatographic retention time (Fig. S13). Therefore, for the isotopologue containing 10 deuterons the effect of isotopic substitution on the retention time cannot be neglected. Application of fragmentation techniques combined with liquid chromatography ambiguously confirmed the sequences of analyzed modified peptides (Fig. S14).

The results indicate, that base catalyzed H/D exchange undergoes only if N-terminal amino group is modified. However only ε-amino groups of Lys reacts with high yield with pyrylium salts. Unfortunately, protons in modified side chain of lysine are not susceptible to H/D exchange. On the other hand, pyrylium salts substituted in aromatic rings by deuterium give high mass shift, beneficial for analysis of large peptides with isotopic dilution method, but large number of deuterons (10) results in detectable shift in retention time of modified peptides, which may be not convenient in quantitative analysis. The new isotopologues of pyrylium salts labeled with stable isotope ^13^C are currently tested in our group.

### Analysis of detection limit of modified peptides

The detection limit for peptides modified with pyrylium salts was determined using two model compounds: TPP^+^-Gly^#^-Leu-OH and TMP^+^-Gly^#^-Leu-OH. The experiment was carried out on Shimadzu LCMS-8050 mass spectrometer equipped in triple quadrupole mass analyzer using MRM (Multiple Reaction Monitoring) mode. To achieve the maximum sensitivity, an automatic MRM optimization method was performed as well as the optimization for needle setting in electrospray (ESI) ion source. The samples were prepared using serial dilution technique. The known quantity of a peptide was dissolved in 1 ml of mixture of water: acetonitryle (50:50) containing 0.1% formic acid and then it was diluted using the same mixture in silicon-coated Eppendorf tubes in order to avoid analyte adsorption on a vessel wall. We injected 1 μl of a solution containing a known concentration of analyte directly to the mass spectrometer in a flow injection mode). Thus, basing on these data we calculated the quantity of a peptide used for analysis. MRM analysis was performed for TPP^+^-Gly^#^-Leu-OH using 479.2 > 308.2* m/z* as a transition pair and 479.20 > 320.10 *m/z* as a second transition pair. The optimum collision energy during the measurement was 41 eV. For the peptide TMP^+^-Gly^#^-Leu-OH, we also used two transition pairs in MRM mode: 292.80 > 122.10* m/z* and 292.80 > 135.15 *m/z.* The collision energy obtained for these transitions pairs after automatic optimization was 34 eV. The detection limits were 1 and 3 attomoles for TPP and TMP-derivatized peptides, respectively. (Fig. S15). These experiments demonstrated that efficiencies of both reagents used as enhancers of peptide ionization are comparable; however a slightly better sensitivity caused by derivatization using TPP is consistent with the anticipation due to a higher hydrophobicity of the phenyl substituent.

### Derivatization of complex mixtures of peptides in solution

Results presented above confirmed that 2,4,6-trisubstituted pyrylium salts efficiently derivatized peptides on a solid support and that obtained conjugates have a high ionization efficiency. The fragmentation patterns of the derivatized peptides indicated, that at low and moderate collision energy produced abundant y type ions allowing convenient sequencing, while at high collision energy spectra were dominated by protonated 2,4,6-triphenyl and 2,4,6-trimethyl pyridinium ions that are suitable as reporter ions in MRM analysis. To test the ability of pyrylium salts to modify peptide amino groups in solution the experiments were performed on the mixture of amino acids, two combinatorial peptide libraries and enzymatic hydrolysates of model proteins, such as ubiquitin and bovine serum albumin (BSA). In most cases excess of 2,4,6-trisubstituted pyrylium salt was necessary to ensure quantitative derivatization of amino groups. Only the mixture of amino acids was treated with the equimolar amount of pyrylium salt to compare the affinity of the reagent to particular amino acids. Derivatization products were analyzed by LC-MS since direct measurement was not efficient because of the ionization suppression caused by excess of pyrylium salt.

The derivatization of the amino acids mixture (His, Arg, Lys, Phe, Ile, Trp, βAla, Met, Pro, Cys, Asn, Val, Gly, Ser, Gln, Tyr, Asp, Glu and Thr) was optimized using different solvents and applying the excess of triethylamine and acetic acid. We performed experiments in DMF solution which was applied previously during experiment on solid support as well as in various solvents compatible with MS analysis, including THF, MeOH and MeCN and their mixtures. The best results were obtained using the mixture of THF/MeCN (1:1) or DMF as solvents with addition of 2 eq of trimethylamine followed by 2 eq of acetic acid. The representative ESI-MS spectrum for the experiment preformed in THF/MeCN is presented in Fig. S16. The most abundant signals correspond to β-Ala (*m/z* 380.165; z = 1) and Gly (*m/z* 366.149; z = 1), respectively. Additionally, two amino groups in lysine residue were modified (*m/z* 364.169; z = 2). The signals of other modified amino acids were characterized by low intensity (lower than 20% of relative abundance). The signals corresponding to modified Arg, Pro and His were not identified at all. These data confirmed that reactivity of unhindered amino acids (β-Ala and Gly) was higher than of amino acids with an additional substituent on α-carbon atom. As could be expected, the secondary amine in Pro residue is not susceptible to derivatization with pyrylium salts.

### Derivatization of combinatorial libraries of peptides

Peptide combinatorial libraries were prepared according to the standard split and mix method^34^. Their derivatizations were carried out using the protocol applied for the mixture of amino acids (details are given in Experimental section). The crude mixtures obtained by derivatization with 2,4,6-triphenylpyrylium salt of the library dissolved in THF and MeCN were analyzed by LC-MS. Since some peptides were not freely soluble in THF/MeCN mixture, sonication was necessary. We identified 38 derivatized peptides, out of the 45 possible (Table S1). Our results correlate well with reactivities of amino acids evaluated in previous experiment. In all cases where lysine residue was present in the peptide sequence the derivatization took place. The signals corresponding to peptides modified on ε-amino group of lysine were characterized by higher intensity than signals corresponding to peptides derivatized at the N-terminus. The extracted ion chromatograms for all identified peptides in LC-MS experiment are presented in Fig. S17. In a few cases, one molecular mass was represented by two or three chromatographic peaks, corresponding to isomeric compounds (e.g. for signal *m/z* 509.7 two peaks appeared corresponding to products of derivatization of KFSG and FGSK peptides, Fig. S18). The most abundant peaks correspond to lysine-containing peptides with two pyridinium salts attached: *m/z* 509.731; 500.244; 554.754 (Fig. S17). In this case one modification site was located on ε-amino group of lysine residue while the second one on the N-terminal α-amino group. As it was previously noticed the modification does not appear on proline residue. Interestingly, the intensity of signals of N-substituted products was lower in cases where the proline was the second amino acid residue. On the other hand, signals corresponding to modified peptides with N-terminal Phe had unusually high intensity which may be a result of the hydrophobic effect. Representative XIC chromatogram and ESI-MS spectra were presented in Fig. S17. These two peaks in chromatogram correspond to the same molecular mass (Fig. S18). Fragmentation spectra revealed the characteristic ion at 308 *m/z* in both cases (Fig. S19).

### Derivatization of ubiquitin hydrolysate

To test the applicability of the proposed method to the analysis of protein hydrolysates we performed the derivatization of peptic ubiquitin digest prepared according to the previously described procedure^35^. To increase solubility of the pyrylium salt, the reaction was carried out in DMF, which also enabled a complete dissolving of peptides. The derivatization was performed using an excess of pyrylium salt. The reaction mixture was evaporated to dryness in the steam of nitrogen and the residue after redissolving was subjected to MS and LC-MS analysis. ESI-MS spectrum (Fig. S20) obtained after derivatization using TPP showed that the abundant peaks present on the spectrum correspond to TPP derivatives of peptides containing lysine residue. Depending on the number of lysine residues in the peptide sequence, derivatives containing one, two or three ionization markers were detected. It should be noted, that the excess of reagent applied to quantitatively derivatize peptides resulted in strong peak at *m/z* 309.12 corresponding to the pyrylium salt, which resulted in suppression of modified peptides signals.

**Figure 5:**
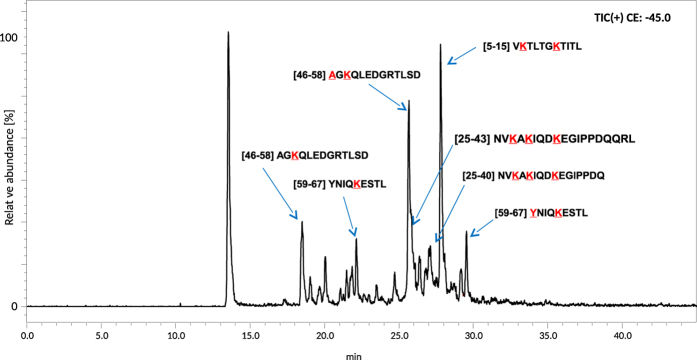
LC-MS analysis of derivatized peptic digest of ubiquitin (precursor ion scan).

**Figure 6:**
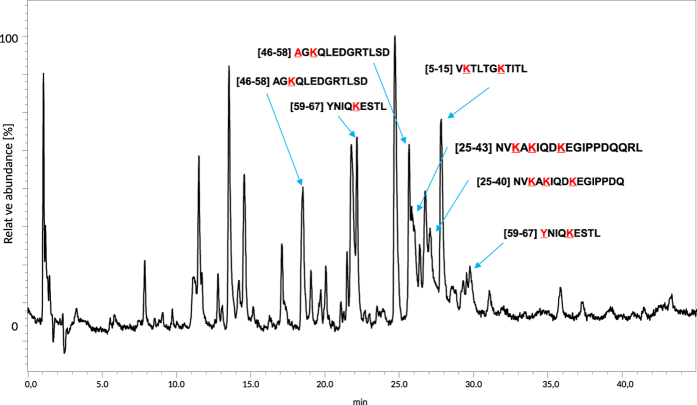
LC-MS analysis of derivatized peptic digest of ubiquitin **(Q1Q3 scan)** (amino acid residues marked with red colour were modified with TPP moiety).

We have demonstrated above, that in CID experiment 2,4,6-triphenylpyridinium salts generate abundant 2,4,6-triphenylpyridinium cation (at *m/z* 308.132), therefore such derivatized peptides can be detected by LC-MS in precursor ion mode using triple quadrupole instrument. This experiment was performed on a derivatized ubiquitin peptic digest according to the procedure described in Experimental section. The precursor ion chromatogram obtained is presented in Fig. 5. The retention times of species found in this chromatogram correspond to these on chromatogram obtained in full scan mode (Fig. 6). The subsequent analysis based on LC-MS results revealed full coverage in respect to fragments containing lysine residues (6 K, 11 K, 27 K, 29 K, 33 K, 63 K), which in fact covers 67% of the protein sequence (Fig. S21). On the other hand, the peptides which does not contain lysine moieties were not derivatized and consequently not detected as substituted pyridinium derivatives. The list of identified peptides is presented in Table S2. All lysine residues in ubiquitin molecule were modified, independently whether the peptide sequence has one lysine residue as in a case of YNIQKESTL [59–67] or three residues as in [25–40] NVKAKIQDKEGIPPDQ peptide fragment. We observed only two peptides in which the TPP moiety was attached to N-terminal α-amino group (AGKQLEDGRTLSD [46–58] and YNIQKESTL [59–67] peptides) which additionally confirms lower reactivity of α- in comparison to ε-amino group. The identity of detected peptides was confirmed by additional LC-MS experiment on time of flight (micrOTOF-Q) mass spectrometer performed to obtain high-resolution mass spectra. The measured and calculated monoisotopic masses of peptides are presented in Table S2. Extracted ion chromatograms for all identified modified peptides are presented in Fig. S22. The two most abundant signals correspond to peptide [59–67] for the ion at *m/z* 838 and [46–58] for the ion at *m/z* 560.

We also have performed additional experiment on the derivatization of peptic digest of ubiquitin. Two equal amounts of ubiquitin – standard protein and ubiquitin fully labeled with ^13^C isotope were subjected to pepsin catalysed hydrolysis. Then sample obtained by the hydrolysis of protein with natural isotopic composition was additionally incubated with triphenylpyrylium salt. Both samples were combined and subjected to LC-MS analysis. All procedures were performed according to Experimental Section.

**Figure 7:**
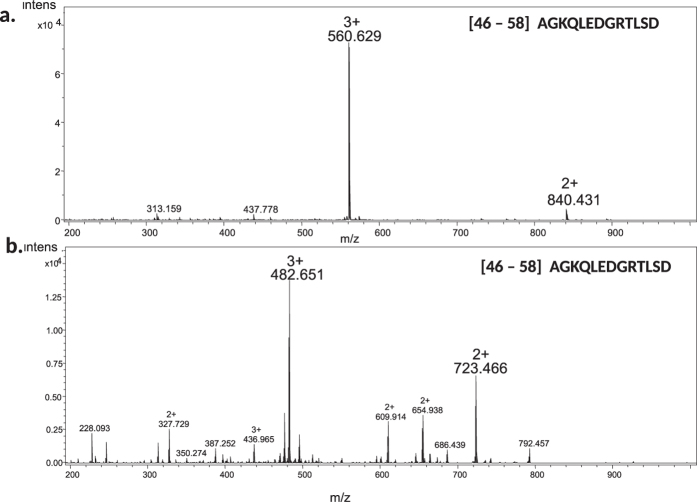
The comparison of ionization efficiency of selected peptide obtained from peptic digest of ubiquitin: (**a**) containing TPP^+^ tag b. without ionization tag (peptide labeled with ^13^C isotope) The experiment was performed in LC-MS mode. The given mass spectra corresponds to two chromatographic peaks. The spectrum presented in panel a corresponds to the AGKQLEDGRTLSD peptide tagged at Lys moiety, while the spectrum in panel (**b**) – to the unmodified peptide. The details of experiment were given in Results and Discussion.

This approach simultaneously provides information on the level of derivatization of peptides and increase in the abundances of signals of particular peptides resulting from chemical modification. These results indicate, that the yield of modification by pyrylium salt is practically 100% (MS spectrum reveals the presence only peptides containing ^13^C isotope – derived from ubiquitin not treated with pyrylium salt, but not peptides with normal isotopic composition). On the other hand the same experiment allows direct comparison of the intensity of peak corresponding to ^13^C substituted peptide (from from the peptic hydrolysis of ^13^C substituted ubiquitin) and peptide with normal isotopic composition modified with pyrylium salt. Example of spectrum in presented in the Fig. 7. The increase in the abundance of peptide peak resulting from the pyrylium salt treatment is in the range 4–10 times.

### Derivatization of BSA hydrolysate

The derivatization with 2,4,6-triphenylpyrylium salt was also tested on more complex system such as the tryptic BSA digest. The experiment was carried out on a commercially available BSA standard hydrolysate (reduced and alkylated with iodoacetamide). The reaction between sample and pyrylium salt was performed using the protocol described above for ubiquitin. After derivatization the sample was analyzed by LC-MS (triple quadrupole mass spectrometer) which enabled the analysis both in a precursor ion scan and a full scan mode. The measurement using the precursor ion scan mode is very informative since it allows selective detection of modified peptides. The obtained chromatogram revealed many peaks with the retention time from 20 to 35 min (Fig. S23). Basing on ExPASy Bioinformatic Resource Portal^36^ and UniProtKB^37^ database a theoretical list of peptides formed after tryptic digestion of BSA was generated. These mases were used to obtain the list of *m/z* values for derivatized peptides. An additional LC-MS experiment using TOF mass spectrometer was performed to obtain the accurate monoisotopic masses and to compare them with these calculated theoretically. The molecular masses found in the spectra and calculated ones are presented in the Table S3. Control LC-MS analysis for unmodified BSA digest was performed. Similarly, the molecular masses found in the spectra and calculated are presented in the Table S4. As we presumed, most of signals corresponded to peptides containing lysine residue on C-terminus due to its higher susceptibility to this modification. Thus, most of peaks were double charged because of the presence of the fixed charge on the nitrogen atom and the addition of proton to the free amine group, however, in some cases, the charge (2+) resulted from the presence of two TPP attached to peptides. The first one was attached to ε-amine group of lysine residue and the second to N-terminus. The analysis of high-resolution spectra allowed confirmation of the identity of eighteen modified peptides which corresponds to 48% of peptides containing Lys moiety (Fig. S24). On the other hand, the analysis of unmodified tryptic digest of BSA performed at the same conditions revealed presence of 12 peptides. It should be noted, that only peptides containing Lys residues were susceptible to this chemical modification. The tryptic peptides with C-terminal Arg instead of Lys are poorly derivatizable since N-terminal amino group reaction with pyrylium salt is less efficient than modification of Lys side chain. Only one signal corresponding to a peptide with arginine on C-terminus and modified N-terminal amino group of serine residue was present in the spectrum (SEIAHR [29–34]).

Wasslen and Co.^38^ reported that AEFVEVTK [248–256], a tryptic fragment of BSA was not detectable in MS experiment as an unmodified compound, probably due to its low ionization efficiency or low proton affinity. However, the authors reported, that upon a prior derivatization *via* trimethylation of lysine, this fragment was detected during MRM experiment. Our study revealed the presence of the discussed peptide in LC-MS experiment as triphenylpyridinium derivative but not as an unmodified peptide which illustrates ionization enhancement resulting from modification of tryptic peptides with pyrylium ion (Fig. S25).

Data reported in literature on the effect of modification of peptides with ionization tags are numerous but not very consistent. The improvement in abundance of peptide peak resulting from chemical modification depends on the ionization tag and on the peptide sequence. Values reported in literature vary from 3 to 40, however there are a few reports claiming 100 times increase in the signal intensity^39^,^40^,^41^. The ionization tags reported herein increase the signal intensity approx. 10 times, although for certain sequences the increase in intensity up to 100 fold was observed. The sequence coverage reported for reagents used for modification of tryptic peptides (DiLeu and iTRAQ reagents) was approximately 43%^42^. The sequence coverage in our studies is similar to this value.

The yield of chemical modification of peptides present in enzymatic digest with pyrylium salts is quantitative. However, other reagents reported in literature may also modify peptides in almost quantitive way.

The advantage of pyrylium salts as reagents for ionization tagging of peptides lies in high regioselectivity of these reagents, the fragmentation patterns of labeled peptides and possibility of convenient and economic incorporation of isotopic label.

## Conclusions

We designed and synthesized new ionization tags: 2,4,6-triphenylpyridinium and 2,4,6-trimethylpyridinium salts. New ionization tags based on 2,4,6-trialkylpyridinium scaffold are promising signal enhancers for qualitative peptide analysis. The MS/MS spectra of tryptic peptides derivatized by pyridinium on ε-amino group of C-terminal lysine side chain show dominant *y*-type ions, allowing facile sequencing of peptides and provide full sequence coverage during fragmentation experiment. Synthesis of deuterated isotopologues is simple, which makes these tags suitable for isotopic dilution quantitative analysis of primary amines. LC-MS analysis, especially in precursor ion scan mode, provides a convenient tool for a rapid detection of pyridinium modified peptides, which significantly facilitates data processing. A new approach basing on pyrylium salts does not require additional activation of ionization enhancer, since pyrylium salts are itself reactive toward amino groups. Moreover these reagents are selective toward ε-amino group of lysine, which makes this approach particularly useful for tryptically digested proteins containing lysine residue on C-terminus. On the other hand, pyrylium salts exhibit relatively low reactivity in respect to α-amino groups. Even though in model conditions (results obtained for combinatorial libraries), derivatization of N-terminal amino groups is possible, the reaction of this reagent with enzymatic digests results in chemical modification almost exclusively ε-amino groups of Lys residues. Therefore, the proposed approach has limitations since tryptic peptides with Arg residues would be excluded from labeling reactions and subsequent quantitative analyses.

The modification with pyrylium salts allows peptide detection at attomol level. The relative simplicity of derivatization technique, commercial availability of pyrylium salts and their low price makes described technique a promising tool for protein analysis.

## Experimerntal Section

Other details concerning used reagents in synthesis and applied equipment are placed in Supporting Information.

### Synthesis 2,4,6- trisubstitutedpyrylium trifluoromethansulphonate

#### Synthesis of benzalacetophenone

The synthesis was performed according to the procedure described by Kohler^43^ with small modifications. 20 ml of 10% NaOH, 12 ml of ethanol and 4.8 g (40 mmol) of acetophenone was placed in the Erlenmayer flask. 4.2 g (40 mmol) of benzaldehyde was added to the stirred mixture. The resulting mixture was stirred for 70 min maintaining the temperature of 25–30 °C. After precipitation of the product, the Erlenmayer flask was cooled for 30 min in the ice bath in order to further precipitation. The obtained crystals were washed with cold water until the washings have reached the pH 7, followed by washing with cold ethanol.

Yield 70%, ESI-HR-MS: *m/z* found 231.078, calculated for (C_15_H_12_O+Na)^+^ 231.078, m.p. 57 °C (literature m.p. 55–57 °C).

#### Synthesis of 2,4,6-triphenylpyrylium trifluoromethansulphonate

3 g (14.4 mmol) benzalacetophenone, 0.87 g acetophenone (7.2 mmol) and 3 ml of dichlomethane was placed in a three-necked round bottom flask, equipped with a thermometer and a dropping funnel. The content of flask was heated and then 2 ml of freshly distilled trifluoromethansulphonic acid (TFMSA) was added dropwise for 30 min. After addition of first portion of TFMSA, the mixture becomes orange changing to brownish yellow at the completion of reaction. The mixture was heated under reflux for 1 hour. After that time it was cooled to room temperature and 30 ml of diethyl ether was added resulting in precipitation of bright yellow crystals. The obtained suspension was allowed to stand overnight in refrigerator. The precipitate was subsequently collected on a Shott funnel and dried *in vacuo.*

Yield 50%. ESI-HR-MS: *m/z* found 309.120, calculated for (C_23_H_17_O)^+^ 309.127, HPLC: Rt (retention time): 22.6 min (conditions for HPLC are given in “Experimental” section) m.p. 253 °C.

#### Synthesis of 2,4,6-trimethylpyrylium trifluoromethansulphonate

18 ml (192 mmol) of acetic anhydride and 3.2 ml (34 mmol) of anhydrous *tert*-butyl alcohol was placed in the Erlenmayer flask. The next step consisted of addition of 3 ml (3.3 mmol) of trifluoromethansulfonic acid in a room temperature for a period of 10 min. A vigorous stirring was required for heat dissipation. After that time the mixture was cooled in ice bath followed by addition of 100 ml of diethyl ether, which resulted in precipitation of yellow crystals. The precipitate was filtered off, washed by diethyl ether and air-dried.

Yield: 50%. ESI-HR-MS: *m/z* found 123.080 calculated for (C_8_H_11_O)^+^ 123.088, HPLC: Rt (min): 10.2 (conditions for HPLC are given in “Experimental” section, Fig. S1 Supporting Information), m.p. 243 °C (literature m.p. 244 °C).

### Synthesis of 2,4,6-triphenylpyrylium trifluoromethansulphonate-d_10_

#### Synthesis of acetophenone-d_10_

7.5 g finely pulverized AlCl_3_ (5.6 mmol) and 10 g of deuterated benzene were placed in two-necked round bottom flask equipped with a thermometer, dropping funnel, reflux and magnetic stirrer. The flask was cooled with a cold water (do not use ice bath - the risk of solidifying of benzene). The next step involved addition of 2.4 ml (25 mmol) of acetic anhydride for a period of 30 min, after that the mixture was allowed to stirr for the another 30 min with heating in a boiling water bath. The completion of the reaction was determined by the disappearance of hydrogen chloride secretion. The content of the flask was cooled, poured into the mixture of a crushed ice (20 g) with sulphuric acid (20 ml) and stirred until the catalyst decomposition. The obtained mixture was transferred to a separatory funnel and extracted twice by 10 ml of diethyl ether. The combined organic layers were washed with 10 ml of 10% NaOH followed by demineralized water. The ethereal extract was dried using anhydrous MgSO_4_.

Yield: 70%, ESI-HR-MS: *m/z* found 126.096, calculated for (C_8_H_3_D_5_O+H)^+^ 126.096.

#### Synthesis of benzalacetophenone-d_5_ and 2,4,6-triphenylpyrylium trifluoromethansulphonate-d_10_

The entire route of synthesis for the title compounds were performed according to the procedure described above including the usage of deuterated acetophenone.

HR-MS: *m/z* found 214.122 calculated for (C_15_H_8_D_5_O)^+^ 214.127, HPLC: Rt (min): 12.5 (conditions for HPLC are given in “Experimental” section, Fig. S2 Supporting Information), HR-MS: *m/z* found 319.183, calculated for (C_23_H_7_D_10_O)^+^ 319.190, HPLC: Rt (min): 23.1 (conditions for HPLC are given in “Experimental” section).

### Synthesis of model modified peptides on solid support/ Derivatization on support

All models peptides were synthesized manually according to the standard Fmoc strategy using TBTU as a coupling reagent^44^. *More details are placed in Experimental Section in Supporting Information*.

### H/D exchange

The hydrogen-deuterium exchange on α-carbon of peptides containing 1-(carboxymethyl)pyridin-1-ium group was carried out according to the procedure developed in our research group for betaine derivatives^45^. The derivatized peptide containing 1-(carboxymethyl) 2,4,6-trimethylpyridinium and 1-(carboxymethyl) 2,4,6-triphenylpyridinium residus on the N-terminus were dissolved in D_2_O containing 1% TEA. After 10 min incubation at room temperature products were lyophilized and then subjected to back-exchange by dissolving in a mixture of H_2_O/MeCN/HCOOH (50:50:0.1). H/D exchange of hydrogens of methyl groups in TMP^+^ derivatized peptides was conducted in conditions given above for a period of 24 h in 50 °C.

### Synthesis of combinatorial libraries of peptides

Tetrapeptides libraries were synthesized on solid support according to the standard Fmoc protocol and “split and mix” strategy^34^. In order to obtain the library 1, three Wang resins: Fmoc-Gly-Wang, Fmoc-Phe-Wang, Fmoc-Lys(Boc)-Wang with the loading of: 0.61, 0.63, 0.63 mmol/g, respectively, were used. These combined resins were subjected to serine residue coupling, so that this position of peptide sequence has been fixed. Then, the mixture of resins beads was divided into three parts and transferred to syringe reactors separately. Further amino acid residue coupling was carried through addition of Fmoc-Gly-OH, Fmoc-Phe-OH, Fmoc-Pro-OH to three separate reactors. After the completion of reaction the resin beads were combined and then divided again, this time into five parts, whereby the next coupling using Fmoc-Ile-OH, Fmoc-Lys(Boc)-OH, Fmoc-Phe-OH, Fmoc-Leu-OH, Fmoc-Val-OH in separate syringes was performed. The synthesis of the library 2 was based on the same strategy involving the usage of other amino acids residues. The obtained peptides sequences are shown in the Results section. The obtained mixture of peptides (Library 1 and Library 2) was subjected to ESI-MS analysis to confirm the composition of the mixture (the number of peptides) before derivatization.

### Solution-Phase derivatization of peptides

#### Derivatization of peptides library

In a 1.5 ml Eppendorf vial 0.2 mg of peptides library was dissolved (assisted with sonication) in 100 μl MeCN/THF (50:50) solution containing 0.18 mg (0.45 μmol) of 2,4,6-triphenylpyrylium salt and the equivalent amount (0.45 μmol) of TEA. After 20 min of incubation at room temperature, an equivalent amount (0.45 μmol) of acetic acid was added. The derivatization was proceeded for 3 h. After the completion of reaction, the sample was evaporated to dryness and lyophilized.

#### Derivatization of ubiquitin hydrolysate

0.1 mg of peptic digested ubiquitin^35^ was dissolved in 100 μl DMF solution containing 0.25 μmol of 2,4,6-triphenylpyryllium salt and 0.25 μmol of TEA (as a solution in DMF). After 20 min, an equivalent amount (0.25 μmol) of acetic acid was added and allowed to react for 4 h. At the end the sample was evaporated under gentle stream of nitrogen and lyophilized.

#### Derivatization of Bovine Serum Albumin (BSA) hydrolysate

250 pmol of tryptically digested BSA was dissolved in 100 μl of DMF. Then, it was added 40 μl (100 nmol) of 2,4,6-triphenylpyrylium salt solution in DMF (1 mg/ml) and the equivalent amount of TEA (as a solution in DMF). After 20 min, the same amount of acetic acid was added. The sample was allowed to react for 4 h and then evaporated to dryness and lyophilized.

#### LC-MS

The LC-MS analysis was performed on Shimadzu LCMS-8050 equipped with a triple quadrupole mass spectrometer and on Agilent 1200 HPLC system coupled to a micrOTOF-Q system mass spectrometer (Bruker, Daltonics, Germany). LC-MS analysis on Shimadzu LCMS-8050 was carried out using precursor ion mode as well as Q1Q3 scan. For the experiment using precursor ion mode an fragmentation ion at *m/z* 308.2 (2,4,6-triphenylpyridinium cation) and the collision energy of 45 eV were chosen. Separation was carried out on an RP-Zorbax (50 × 2.1 mm, 3.5 μm) column with a gradient elution of 5–60% B in A (A, 0.1% HCOOH in water; B, 0.1% HCOOH in MeCN) at room temperature over a period of 45 min (flow rate: 0.1 ml/min). The samples of derivatized hydrolysate were dissolved in 400 μl of water:acetonitrile mixture. The injection volume was: 4 μl for BSA and 1 μl for ubiquitin hydrolysate (Shimadzu LCMS-8050). The alternative LC-MS experiment on Agilent 1200 - micrOTOF-Q system was performed using the same separation conditions. The injection volume was 5 μl for all protein hydrolysates.

## Additional Information

**How to cite this article**: Waliczek, M. *et al*. Peptides Labeled with Pyridinium Salts for Sensitive Detection and Sequencing by Electrospray Tandem Mass Spectrometry. *Sci. Rep.* **6**, 37720; doi: 10.1038/srep37720 (2016).

**Publisher's note:** Springer Nature remains neutral with regard to jurisdictional claims in published maps and institutional affiliations.

## Supplementary Material

Supplementary Dataset 1
