# Supplementary material for: Peptides Labeled with Pyridinium Salts for Sensitive Detection and Sequencing by Electrospray Tandem Mass Spectrometry
Source: Sci Rep. 2016 Nov 28;6:37720. doi: 10.1038/srep37720 (PMC5125270; doi:10.1038/srep37720)
Supplement: Supplementary Dataset 1 [file srep37720-s1.doc]

**PEPTIDES LABELED WITH PYRIDINIUM SALTS FOR SENSITIVE DETECTION AND SEQUENCING BY ELECTROSPRAY TANDEM MASS SPECTROMETRY**

Mateusz Waliczek, Monika Kijewska, Magdalena Rudowska, Bartosz Setner,

Piotr Stefanowicz*, Zbigniew Szewczuk

*Faculty of Chemistry, Wroclaw University, Wroclaw, Poland*

*F. Joliot-Curie Street 14, 50-383 Wroclaw*

**piotr.stefanowicz@chem.uni.wroc.pl*

**EXPERIMENTAL SECTION**

*Reagents:*

Solvents for peptide synthesis (analytical grade) were obtained from Sigma-Aldrich (dimethylformamide-DMF; trifluoroacetic acid-TFA) and J. T. Baker (methanol). All amino acids derivatives were purchased from Iris Biotech GMBH. Solvents for LC-MS and MS measurements: acetonitrile (MeCN), methanol (MeOH), formic acid (HCOOH) were purchased from Sigma Aldrich: . Reagents and solvents used in pyryllium salts were obtained from: Eurochem BGD (NaOH, AlCl_3_), POCh Basic (ethanol), Chempur (diethyl ether and dichloromethane-DCM), VEB Jenapharm-Laborchemie APOLDA (acetophenone), Sigma-Aldrich (benzaldehyde, *tert*-buthyl alcohol), Fluka AG (trifluoromethanesulfonic acid), Lachner (acetic anhydrite). Reagents for derivatization reactions were purchased from: J.T Baker (acetic acid) and Sigma-Aldrich (trimethylamine-TEA; tetrahydrofuran - THF). Tryptic digested bovine serum albumin (BSA) was purchased from BioLabs. Ubiquitin from bovine erythrocytes was obtained from Sigma-Aldrich. Recombinant 13C labeled ubiquitin was purchased from ASLA Biochem (Latvia). Silicon-coated Corning Costar Eppendorf tubes were purchased from Sigma-Aldrich.

*Equipment:*

*Mass spectrometry analysis*

Mass spectrometric measurements were performed on a quadrupole time-of-flight instrument (micrOTOF-Q, Bruker, Germany) equipped with an electrospray (ESI) source. Samples containing 10-100 μg of an analyte were introduced using direct injection to mass spectrometer with a flow rate of 3 μl/min. The potential between the spray needle and the orifice was set to 4.5 kV. The micrOTOF-Q instrument equipped with an ESI source with ion funnel, was operated in the positive or negative ion mode and calibrated before each analysis with the Tunemix mixture (Agilent) by a quadratic method. In MS/MS experiments, the collision energy (10–60 eV) was optimized for the best fragmentation of a peptide. The measurements were carried out in the mixture of solvents consisting of acetonitrile:water:formic acid (50:50:0.1). In the MS/MS mode, the quadrupole was used to select precursor ions, which were then fragmented in the hexapole collision cell generating product ions that were subsequently mass analyzed by the orthogonal reflectron TOF mass analyzer. Argon was used as a collision gas.

*HPLC*

HPLC separation of intermediates and synthesis products of pyrylium salts were carried out on Thermo Separation HPLC system with a UV detection (210 nm) and a Vydac Protein RP C18 column (4.6 × 250 mm, 5 μm), with a gradient elution of 0%–80% S2 in S1 (S1 = 0.1% aqueous TFA in H_2_O; S2 = 80% acetonitrile + 0.1% TFA) for 40 min (flow rate 1 mL/min).

*Synthesis of model modified peptides on solid support/ Derivatization on support*

After the synthesis of peptide the protecting group was removed from N-terminus using 25% piperidine in DMF. The modification was incorporated according the protocol: 3 equivalents of 2,4,6-triphenylpyrylium salt and 3 equivalents of triethylamine (TEA) in DMF were added to peptide attached to the Resin. After 20 min, 3 equivalents of acetic acid were added and then the mixture was allowed to stir for 3 h. Finally, the resin was washed 5 times with DMF in order to remove the remains of derivatizing agent and then 3 times with dichloromethane and 4 times using methanol. The last step consisted of drying of a resin in vacuum dessicator. The peptide was cleaved from resin using mixture of trifluoroacetic acid : water : triisopropylsilnae (95 : 2.5 : 2.5; v : v : v) over the time of 2 h. The solution was evaporated under gentle stream of nitrogen and then lyophilized.

**Fig. S1** Chromatogram of benzalacetophenone-*d_5_*

**Fig. S2** Chromatogram of 2,4,6-trimethylpyrylium trifluoromethansulphonate

**Fig. S3** Mechanism of the reactions of primary amines with pyrylium cations

**Fig. S4** ESI-MS spectrum and chromatogram of 2,4,6-trimethylpyrylium salt (TPP)

**Fig. S5** ESI-MS of modified peptide TPP^+^-Gly^#^-Phe-OH (Gly^#^ - glycine residue without the amino group)

**Fig. S6** ESI-MS/MS spectrum of TPP^+^-Gly^#^-Phe-OH. The proposed structures of the fragment ions are presented

**Fig. S7** ESI-MS/MS spectrum of modified peptide TMP^+^-Gly^#^-Phe-OH

**Fig. S8** ESI-MS and chromatogram of 2,4,6-triphenylpyrylium trifluoromethansulphonate*-d_10_* (TPP)

**Fig. S9** ESI-MS spectra of TPP^+^-Gly^#^-Ala-Phe-Gly-NH_2_ and its isotopologue (TPP^+^-Gly^#^-Ala-Phe-Gly-NH_2_-d_10_)

**Fig. S10** ESI-MS spectra of TMP^+^-Gly^#^-Phe-OH before and after incubation D_2_O/TEA in 50ᵒC for 24h

**Fig. S11** ESI-MS/MS for TMP^+^-Gly^#^-Phe-OH and its deuterated analogues

**Fig. S12.** “Peak purity analysis”

A) LC-MS analysis for the compounds TPP^+^-Gly^#^-Ala-Phe-Gly-NH_2_ and TPP^+^-Gly^#^-Ala-Phe-Gly-NH_2_-d_2_; total ion current (red line) and extracted ion chromatograms for the ions 640.3 and 642.3 corresponding to TPP+-Gly^#^-Ala-Phe-Gly-NH_2_ and TPP^+^-Gly^#^-Ala-Phe-Gly-NH_2_-d_2_ respectively; B) ESI-MS spectrum for compounds eluted at different retention times

**Fig. S13** LC-MS analysis for the compounds TPP^+^-Gly^#^-Ala-Phe-Gly-NH_2_ and TPP^+^-Gly^#^-Ala-Phe-Gly-NH_2_-d_10_ A) Extracted ion chromatograms for the ions at 640.3 *m/z* and 650.4 *m/z* corresponding to TPP^+^-Gly^#^-Ala-Phe-Gly-NH_2_ and TPP^+^-Gly^#^-Ala-Phe-Gly-NH_2_-d_10_ respectively; B) ESI-MS spectrum for compounds eluted at retention time 29.3-29.8

**Fig. S14** LC-MS/MS analysis for the compounds TPP^+^-Gly^#^-Ala-Phe-Gly-NH_2_ and TPP^+^-Gly^#^-Ala-Phe-Gly-NH_2_-d_10_ A) Total ion current for fragmentation analysis (red line) and extracted ion chromatograms for the ions at 640.3 *m/z* and 650.4 *m/z* corresponding to TPP^+^-Gly^#^-Ala-Phe-Gly-NH_2_ and TPP^+^-Gly^#^-Ala-Phe-Gly-NH_2_-d_10_ respectively; B) ESI-MS/MS spectrum for ion 650.35 corresponding to TPP^+^-Gly^#^-Ala-Phe-Gly-NH_2_-d_10_; C) ESI-MS/MS spectrum for ion 640.29 corresponding to TPP^+^-Gly^#^-Ala-Phe-Gly-NH_2_

**Fig. S15** MRM analysis for A) TPP^+^-Gly^#^-Leu-OH using two transition pairs: 479.2 > 308.2 *m/z* and 479.20 > 320.10 *m/z;* B) for TMP^+^-Gly^#^-Leu-OH using two transition pairs: 292.80 > 122.10 *m/z* and 292.80 > 135.15 *m/z*.

**Fig. S16** ESI-MS spectrum of the mixture of amino acid residues modified with 2,4,6-triphentlpyryllium salt (^#^ - amino acids without amino, Lys^##^ - amino acid without amino groups)

**Fig. S17** Extracted ion chromatograms of 20 identified signals

**Fig. S18** A) Extracted ion chromatogram for the ion at 509.7; B) ESI-MS spectrum for modified peptide eluted at 38.2-38.3; C) ESI-MS spectrum for modified peptide eluted at 39.8-40.0 (^#^ - amino acids without amino group, Lys^##^ - N-terminal amino acid without amino groups)

**Fig. 19** A) Extracted ion chromatogram for the ion at 509.7; B) ESI-MS/MS spectrum for modified peptide at ion 509.7 *m/z* eluted at 38.2-38.3; C) ESI-MS/MS spectrum for modified peptide at ion 509.7 *m/* eluted at 39.8-40.0 (^#^ - amino acids without amino group, Lys^##^ - N-terminal amino acid without amino groups)

**Fig. S20** ESI-MS spectrum of the derivatized peptic ubiquitin fragments (crude mixture injected directly to mass spectrometer). The Lys residues in sequences are marked in red color (K – denotes lysine residue derivatized by one TPP molecule)

MQIFV**K**TLTG**K**TITLEVEPSDTIENV**K**A**K**IQD**K**EGIPPDQQRLIFAG**K**QLEDGRTLSDYNIQ**K**ESTLHLVLRLRGG

**Fig. S21** Sequence coverage of ubiquitin

**Fig. S22** Extracted ion chromatograms for all identified modified peptide of ubiquitin hydrolysate

**Fig. S23** LC-MS analysis of derivatized bovine serum albumin hydrolysate **(precursore ion scan)**

MKWVTFISLLLLFSSAYSRGVFRRDTHK**SEIAHR**FK**DLGEEHFK**GLVLIAFSQYLQQCPF

DEHVK**LVNELTEFAKTCVADESHAGCEKSLHTLFGDELCK**VASLRETYGDMADCCEKQEP

**ERNECFLSHKDDSPDLPKLKPDPNTLCDEFKADEKKFWGKYLYEIARRHPYFYAPELLYY**

ANKYNGVFQECCQAEDKGACLLPKIETMREKVLASSARQRLRCASIQKFGERALKAWSVA

RLSQKFPK**AEFVEVTKLVTDLTK**VHKECCHGDLLECADDR**ADLAK**YICDNQDTISSKLK**E**

**CCDKPLLEKSHCIAEVEKDAIPENLPPLTADFAEDK**DVCKNYQEAKDAFLGSFLYEYSRR

**HPEYAVSVLLRLAKEYEATLEECCAKDDPHACYSTVFDKLKHLVDEPQNLIKQNCDQFEK**

LGEYGFQNALIVRYTRKVPQVSTPTLVEVSRSLGKVGTRCCTKPESERMPCTEDYLSLIL

NR**LCVLHEK**TPVSEKVTKCCTESLVNRRPCFSALTPDETYVPK**AEFVEVTK**LFTFHADICTLPDTEKQIKK**QTALVELLK**HKPK**ATEEQLKTVMENFVAFVDK**CCAADDKEACFAVEGPKLVV

STQTALA

**Fig. S24** Sequence coverage of BSA

**Fig. S25** LC-MS analysis: A) Extracted ion chromatograms for the ions at 606.76 *m/z* corresponding to modified fragment of peptide [248-256]; B) ESI-MS spectrum for compounds eluted at retention time 29.3-29.8

**Table S1.** The list of identified peptides derivatized with 2,4,6-triphenyl pyrylium salt

| **sequence** | **Calculated mass** | | **Found mass** | |
| --- | --- | --- | --- | --- |
|  | **[M^#^+TPP]+** | **[M^#^+2TPP]^2+^** | **[M^#^+TPP]+** | **[M^#^+2TPP]^2+^** |
| IGSG | 623.2859 | 457.2014 | 623.270 | NF |
| IGSP | 663.3179 | 477.2174 | 663.308 | NF |
| IGSK | 694.3599 | 492.7384 | 694.349 | 492.733 |
| IFSG | 713.3329 | 502.2249 | 713.320 | NF |
| IFSP | 753.3649 | 522.2409 | 753.350 | NF |
| IFSK | 784.4069 | 537.7619 | 784.393 | 537.752 |
| IPSG | 663.3179 | 477.2174 | 663.308 | NF |
| IPSF | 753.3649 | 522.2409 | 753.350 | NF |
| IPSK | 734.3912 | 512.75405 | 734.382 | NF |
|  |  |  |  |  |
| KGSG | 638.2973 | 464.7071 | 638.291 | NF |
| KGSF | 728.3443 | 509.7306 | 728.330 | NF |
| KGSK | 709.3708 | 500.24385 | NF | 500.239 |
| KFSG | 728.3443 | 509.7306 | 728.330 | NF |
| KFSF | 818.3912 | 554.75405 |  |  |
| KFSP | 768.3756 | 529.74625 | 768.360 | NF |
| KPSG | 678.3286 | 484.72275 | 678.316 | NF |
| KPSF | 768.3756 | 529.74625 | 768.360 | NF |
| KPSK | 749.4021 | 520.2595 | 520.254 | NF |
|  |  |  |  |  |
| LGSG | 623.2864 | 457.20165 | 623.270 | NF |
| LGSF | 713.3334 | 502.22515 | 713.320 | NF |
| LGSK | 694.3599 | 492.7384 | 694.349 | NF |
| LFSG | 713.3334 | 502.22515 | 713.320 | NF |
| LFSF | 803.3803 | 547.2486 |  |  |
| LFSK | 784.4069 | 537.7619 | 784.393 | 537.752 |
| LPSG | 663.3177 | 477.2173 | 623.270 | NF |
| LPSF | 753.3647 | 522.2408 | 753.350 | NF |
| LPSK | 734.3912 | 512.75405 | 734.382 | NF |
|  |  |  |  |  |
| VGSG | 609.2708 | 450.19385 | NF | NF |
| VGSF | 699.3177 | 495.2173 | NF | NF |
| VGSK | 680.3443 | 485.7306 | 680.335 | NF |
| VFSG | 699.3177 | 495.2173 | NF | NF |
| VFSF | 789.3647 | 540.2408 | NF | NF |
| VFSP | 739.349 | 515.23295 | NF | NF |
| VPSG | 649.3021 | 470.2095 | NF | NF |
| VPSF | 739.349 | 515.23295 | NF | NF |
| VPSK | 720.3756 | 505.74625 | 720.360 | NF |
|  |  |  |  |  |
| FGSG | 657.2708 | 474.19385 | 657.269 | NF |
| FGSF | 747.3177 | 519.2173 | 747.302 | NF |
| FGSK | 728.3443 | 509.7306 | 728.330 | 509.728 |
| FFSG | 747.3177 | 519.2173 | 747.302 | NF |
| FFSF | 837.3647 | 564.2408 | NF | NF |
| FFSK | 818.3912 | 554.75405 | 818.378 | NF |
| FPSG | 697.3021 | 494.2095 | NF | NF |
| FPSF | 787.349 | 539.23295 | NF | NF |
| FPSK | 768.3756 | 529.74625 | 768.360 | 529.744 |

NF - not found on the mass spectrum

M# -where # denotes deamino.

**Table S2.** The list of identified modified peptides in ubiquitin hydrolysate

|  | **Sequence** | **[TPP+H]^2+^**  **calc/found** | **[TPP+2H]^3+^**  **calc/found** | **[2TPP]^2^+**  **calc/found** | **[2TPP+H]^3+^**  **calc/found** | **[3TPP]^3+^**  **calc/found** | **[3TPP+H]^4+^**  **calc/found** |
| --- | --- | --- | --- | --- | --- | --- | --- |
| 1. | **[4-15]**  **F V K T L T G K T I T L** |  |  | 634.681/634.685 |  |  |  |
| 2. | **[5-15]**  **V K T L T G K T I T L** |  |  | 877.983/877.998 | 585.658/585.671 |  |  |
| 3. | **[25-43]**  **N V K A K I Q D K E G I P P D Q Q R L** |  |  |  |  |  | 762.638/762.650 |
| 4. | **[25-40]**  **N V K A K I Q D K E G I P P D Q** |  |  |  |  | 884.101/884.123 | 663.327/663.344 |
| 5. | **[46-58]**  **A G K Q L E D G R T L S D** |  | 840.407/840.412 | 560.607/560.612 | 657.310/657.325 |  |  |
| 6. | **[59-67]**  **Y N I Q K E S T L** | 693.343/693.390 |  | 838.397/838.416 |  |  |  |

**Table S3.** The list of identified modified peptides in BSA hydrolysate

|  | **Sequence** | **[M+TPP]^+^**  **calc./found** | **[M+H+TPP]^2+^**  **calc./found** | **[M+TPP+TPP]^2+^**  **calc./found** | **[M+H+H+TPP]^3+^**  **calc./found** | **[M+H+H+TPP]^3+^ calc./found** |
| --- | --- | --- | --- | --- | --- | --- |
| 1. | **ADLAK [280-285]** |  |  | 546.262/549.234 |  |  |
| 2. | **FWGK [156-160]** |  | 414.199/414.177 |  |  |  |
| 3. | **SEIAHR [29-34]** | 1002.51/1002.47 |  |  |  |  |
| 4. | **LVTDLTK [256-263]** |  | 540.294/540.264 |  |  |  |
| 5. | **LCVLHEK [483-489]** |  |  | 740.346/740.355 |  |  |
| 6. | **AEFVEVTK [248-256]** |  | 606.802/606.768 | 751.857/751.813 |  |  |
| 7. | **DLGEEHFK [37-44]** |  | 632.787/632.748 |  |  |  |
| 8. | **QNCDQFEK [413-420]** |  | 651.268/651.287 |  |  |  |
| 9. | **QTALVELLK [552-560]** |  | 652.868/652.828 |  |  |  |
| 10. | **SHCIAEVEK [310-318]** |  |  | 798.357/798.370 |  |  |
| 11. | **LVNELTEFAK [66-75]** |  | 727.374/727.332 |  |  |  |
| 12. | **TCVADESHAGCEK [75-88]** |  |  |  |  | 643.927/643.950 |
| 13. | **HLVDEPQNLIK [392-412]** |  | 798.416/798.373 |  | 532.614/532.585 |  |
| 14. | **EYEATLEECCAK [375-386]** |  |  |  | 598.902/598.886 |  |
| 16. | **ECCDKPLLEK [300-309]** |  | 734.338/734.322 | 879.392/897.368 |  |  |
| 17. | **TVMENFVAFVDK**  **[572-583]** |  | 845.405/845.357 |  |  |  |
| 18. | **DAIPENLPPLTADFAEDK**  **[319-336]** |  | 1123.540/1123.470 |  |  |  |

**Table S4.** The list of identified unmodified peptides in BSA standard digest (CAM- cysteine groups alkylated with iodoacetamide)

|  | **Peptide sequence** | ***m/z* found [*calculated*]** | **charge** |
| --- | --- | --- | --- |
| **1.** | YIC^(CAM)^DNQDTISSK | 722,326 [722,325] | [M+2H]^2+^ |
| **2.** | GAC^(CAM)^LLPK | 758,413 [758,422] | [M+H]^+^ |
| **3.** | YNGVFQEC^(CAM)^C^(CAM)^QAEDK | 874,342 [874,356] | [M+2H]^2+^ |
| **4.** | DDPHAC^(CAM)^YSTVFDK | 777,839 [777,830] | [M+2H]^2+^ |
| **5.** | HLVDEPQNLIK | 653,370 [653,362] | [M+2H]^2+^ |
| **6.** | LKPDPNTLC^(CAM)^DEFK | 788,782 [788,887] | [M+2H]^2+^ |
| **7.** | LVVSTQTALA | 1002,556 [1002,583] | [M+H]^+^ |
| **8.** | SLHTLFGDELC^(CAM)^K | 710,356 [710,350] | [M+2H]^2+^ |
| **9.** | LVNELTEFAK | 582,334 [582,318] | [M+2H]^2+^ |
| **10.** | QTALVELLK | 507,836 [507,813] | [M+2H]^2+^ |
| **11.** | LFTFHADIC^(CAM)^TLPDTEK | 954,435 [954,464] | [M+2H]^2+^ |
| **12.** | DAIPENLPPLTADFAEDK | 978,468 [978,483] | [M+2H]^2+^ |
| **13.** | TVMENFVAFVDK | 700,355 [700,350] | [M+2H]^2+^ |
